# Musculoskeletal education: an assessment of the clinical confidence of medical students

**DOI:** 10.1007/s40037-014-0124-1

**Published:** 2014-05-28

**Authors:** Jeremy Truntzer, Alison Lynch, David Kruse, Michael Prislin

**Affiliations:** 1 University of California, Irvine, CA USA; 2 Present Address: Department of Orthopaedics, Rhode Island Hospital, Brown University, 593 Eddy Street, Providence, RI 02903 USA; 3 Department of Family Medicine, University of California, San Diego, CA USA; 4 Sports Medicine Physician, Orthopaedic Specialty Institute, Orange, CA USA; 5 Department of Family Medicine, University of California, Irvine, CA USA; 6 Department of Family Medicine, Student Affairs School of Medicine, University of California, Irvine, CA USA

**Keywords:** Medical education, Sports medicine, Musculoskeletal education

## Abstract

Musculoskeletal (MSK) conditions account for nearly 15–30 % of encounters in a primary care practice. Yet, studies demonstrate that medical students and residents lack the knowledge and confidence to care for many MSK conditions. This study addresses the design of focused MSK educational practices towards improving students’ knowledge, interest, and confidence for conducting MSK examinations. Students attending a voluntary educational symposium on sports medicine were recruited to participate. The symposium was directed toward teaching elements of the MSK exam. Participants completed validated pre- and post-workshop surveys that assessed confidence in performing MSK examinations as well as satisfaction and perceived importance of MSK education. Additionally, mean survey responses from a convenience group of students who did not participate in the symposium were compared against the intervention group. Thirteen students participated in the educational symposium. Hundred and nine students completed the general survey. In the non-intervention group, students demonstrated knowledge and confidence improvements through the second year of medical school but did not show similar improvement in subsequent years. No difference in MSK confidence scores between fourth-year students going into high versus low MSK focused specialities was observed. In the intervention group students demonstrated improvements in confidence with respect to the knee, shoulder and ankle exams (*p* < 0.01). Areas not covered such as concussions and neuromuscular impairments failed to show significant change. Current core clinical training, at least at our school, does not achieve satisfactory levels of knowledge and confidence with respect to caring for MSK conditions. However, a focused didactic and skill development intervention does produce significant improvements. Follow-up is needed to determine whether these improvements are sustained.

## Introduction

Musculoskeletal (MSK) complaints are among the primary reasons that individuals seek medical attention in the US, comprising 15–30 % of all primary care encounters [1]. With the ageing population, the prevalence of MSK conditions is increasing. The estimated US cost including treatment, diagnosis and lost wages is $848 billion annually, equivalent to 7.7 % of the gross domestic product [2].

However, many current studies have shown that recently graduated medical students and residents lack the clinical knowledge and confidence necessary to care for patients with MSK injuries. Upon administration of an MSK basic competency examination deficiencies were shown at all levels of training [3, 4]. Experts postulate that inadequate time allocated to MSK education and ineffective teaching methods contributes to the problem [5, 6]. For example, only 20.5 % of programmes reported a mandatory MSK clerkship rotation [7]. In response, there has been a national movement to improve MSK education amongst US medical schools [8, 9].

This study addresses the design of MSK education at our institution, specifically the efficacy of focused MSK education towards improving students’ knowledge, interest, and confidence for conducting MSK examinations. An intervention in the form of a pilot programme was designed and implemented to test this model and compared against a general, school-wide survey. We hope our findings may provide a framework for other programmes across the country.

## Materials and methods

This study was reviewed by the institutional review board. In order to gauge the benefit of a small-group, high-impact model at our institution, a pilot project was completed concurrently to a general, school-wide survey. Medical students from all years in training with interests in all specialities were invited to attend a symposium on sports medicine. The symposium consisted of a series of educational lectures and hands-on workshops focused on specific areas of MSK medicine including MSK radiology and physical examination of commonly injured joints.

Those students participating in the intervention (intervention group) were asked to fill out a pre- (baseline) and post-workshop survey following opening and closing sessions of the workshop, respectively. The surveys were modelled after a national survey developed for the evaluation of MSK competency (reported margin of error 3–7 %) [10]. The baseline survey via an online document was also distributed to the larger student body through the school’s official e-mail listing (control group). Surveys were structured based on the following major categories according to an increasing scale from 1 to 5: Confidence in MSK, satisfaction in MSK, perception of MSK importance (Table 1).

**Table 1:** Pre-survey and post-survey responses from the intervention group before and after the symposium. First-year through fourth-year students included

	Pre-survey	Post-survey	*p*-value
Confidence			
How comfortable are you with the musculoskeletal (MSK) exam?	2.0	3.3	<0.001
How comfortable are you treating patients with MSK complaints in general?	1.8	2.8	0.008
How comfortable are you treating patients with osteoporosis and rheumatological complaints?	1.7	2.1	0.28
How comfortable are you treating geriatric patients with MSK pain (ex. knee pain, lower back pain)?	1.8	2.3	0.20
How comfortable are you performing MSK exams on the knee?	2.1	3.5	0.002
How comfortable are you performing MSK exams on the shoulder?	1.9	3.3	<0.001
How comfortable are you performing MSK exams on the ankle?	1.8	3.2	<0.001
How comfortable are you performing MSK exams on the spine?	2.0	2.4	0.28
How comfortable are you treating patients with neuro-muscular deficits?	1.7	2.0	0.44
How comfortable are you treating athletes with sports-related injuries?	1.9	2.5	0.09
How comfortable are you treating patients with concussion?	1.6	1.7	0.60
Satisfaction			
How satisfied are you with the QUANTITY of MSK education in the existing curriculum?	1.7		
How satisfied are you with the QUALITY of MSK education in the existing curriculum?	2.1		
Importance			
How important do you think MSK education is relative to OTHER subjects?	3.7	3.7	0.76

*MSK* musculoskeletal

The Student *t* test was used to compare pre- and post-survey data. Statistical significance was assessed at *p* < 0.05 throughout. Post-hoc power analysis was performed using a two-sample average statistical calculator with 5 % alpha error (Decision Support Systems, Fort Worth, TX).

## Results

Thirteen participants in the intervention group completed pre- and post-surveys. Hundred and nine baseline surveys were received from the control group. The response rate amongst all classes was nearly equal. Average year in medical school (2.2 intervention, 2.4 control) did not differ between the two populations (*p* > 0.05). Further stratifying the populations according to year in training did not yield any pre-survey differences except for confidence in treating concussions. First-year students were considered naive to MSK education, and therefore analyzed separately from subsequent years based on the introduction of MSK education in the second year, when students are exposed to MSK teaching in small-group sessions and community shadowing.

### Confidence in MSK

Results from the control group generated an overall mean confidence score across all MSK topics of 2.8/5 (SD 1.1). No significant difference was found between second-, third- and fourth-year students (*p* > 0.05) for evaluation of the common joints. Students across all years recorded the lowest confidence scores related to the diagnosis and treatment of geriatric patients (2.23, SD 1.1), rheumatological disorders (2.17, SD 1.0), and concussions (1.8, SD 1.1).

Participants in the intervention reported a similar baseline lack of confidence compared with the control. Post-intervention, MSK confidence was statistically increased in the areas of knee (*p* = 0.002), shoulder (*p* < 0.001) and ankle (*p* < 0.001) (Table 1). Areas not covered during the symposium including concussions, geriatrics, neuromuscular diseases, spine, and rheumatology were not significantly changed (*p* > 0.05). Post-hoc power analysis was >80 % in all cases of significant improvement.

Confidence in MSK subjects specifically for fourth-year medical students was stratified according to speciality of interest to determine if anticipated career was a contributing factor. At the time of survey distribution, many fourth-year medical students had already completed a series of clerkships in their fields of interest, which were categorized as having either a major (Orthopaedics, Family Medicine, Paediatrics, PM&R, Emergency Medicine, Neurology) or minor focus (Radiology, Ob/Gyn, Psychiatry, Internal Medicine, Ophthalmology, Pathology, General Surgery) in MSK medicine. Confidence (3.3 Major (SD 1.1) vs. 3.1 Minor (SD 0.8)) was not different between the two groups (*p* > 0.05).

### Satisfaction and importance of MSK training

As scored in the general survey, satisfaction with MSK training showed no significant change across the years in regards to quantity or quality. Moreover, no year averaged a score above adequate (>2.5), with an overall institution score of 2.3 (SD 1.0) and 2.4 (SD 1.1) for quantity and quality as defined in the questionnaire, respectively.

Importance of MSK education in comparison with other subjects received a score of 3.1 across all classes from the general survey. A value of 2.5 was considered average. Amongst students attending the symposium, the importance score (3.7) was unaffected by the intervention.

Respondents were also given the opportunity to comment on the importance of MSK training in an open forum. A few sentiments that represent the majority are provided below:

‘MSK complaints make up a huge proportion of visits, yet I don’t think we get a good foundation. If we had a better curriculum, we’d be saving ourselves a lot of time and headache during our residencies.’: Third-year student.

‘I do not feel comfortable doing the MSK exam because I have not practised it nearly enough.’: Fourth-year student.

## Discussion

### Pilot programme

We attempted to assess the feasibility of teaching MSK medicine in the scope of a pilot programme. We believe that the results from our didactic teaching model accurately reflect the benefit of organized MSK teaching. For example, stations specific for knee, shoulder and ankle assessment generated significant increases in confidence (*p* < 0.01). Whereas areas not covered such as concussions, and neuromuscular-related impairments did not show any change. Therefore, it is more likely that the reported change in confidence is due to a true improvement as opposed to a reporting bias secondary to attendance at the event.

### General survey

Results from the control group suggest that students are aware of the importance of MSK medicine and subsequent value in medical school training. Yet, the students acknowledged a paucity of learning opportunities. For example, despite increased exposure to MSK medicine during clinical years, low satisfaction, quality, and quantity scores remained beyond the second year. We feel the low scores represent a recognized deficiency and concern by students and help validate that exposure alone is not enough to develop satisfactory MSK assessment skills.

The absence of improved confidence scores observed beyond the second year supports the lack of exposure afforded to our students as well as emphasizing the importance of high-yield teaching methods. While small-group teaching modules currently exist during the second year at our institution, MSK exposure during the clinical years is only made available as part of elective courses limited to a small percentage of the student body and briefly during the family medicine rotation. Especially when considering the results from our pilot programme, it is not surprising that confidence scores remained unchanged following the second year when MSK teaching adopts a less rigorous model.

### Limitations

These data are the reflection of a single medical school programme. While we attempted to pool an unbiased set of students through anonymous channels, it is possible that a population with a greater interest in MSK medicine may have completed the survey.

Secondly, our pilot programme was organized around a small sample population in order to provide high-yield teaching from experts in the field. However, we believe the significant changes observed only in the areas covered during the symposium including shoulder, knee and ankle validates the results. Moreover, post hoc power calculations were above 80 % with alpha at 5 % suggesting that the null hypothesis was correctly rejected in spite of smaller sampling.

Thirdly, and importantly, we addressed the success of MSK training based on the level of confidence cited by participants. However, this is a subjective marker and unfortunately does not reflect the true ability of a student to perform the requisite manoeuvers for MSK evaluation. Future evaluations will include the Freedman and Bernstein’s nationally (Freedman 1998) validated basic competency exam in order to elucidate the correlation between competency and confidence in the scope of MSK evaluation.

## Conclusion

As demonstrated in our study, passive teaching alone during the clinical years does not satisfy the necessary level of education to improve confidence in MSK evaluation. Our pilot programme was effective in this regard. We encourage other institutions to implement similar programmes across the country, so as to improve care for patients with MSK conditions.
